# Effects of Metal Foam Insertion on the Performance of a Vacuum Membrane Distillation Unit

**DOI:** 10.3390/membranes15120379

**Published:** 2025-12-13

**Authors:** Nizar Loussif, Jamel Orfi

**Affiliations:** 1 Laboratory for the Study of Thermal and Energy Systems LESTE-LR99ES31, ENIM, University of Monastir, Monastir 5019, Tunisia; loussif.nizare@gmail.com; 2 Higher School of Sciences and Technology of Hammam Sousse (ESSTHS), Department of Physics, University of Sousse, Sousse 4041, Tunisia; 3 Mechanical Engineering Department, King Saud University, Riyadh 11421, Saudi Arabia

**Keywords:** vacuum membrane distillation, metal foam, heat and mass transfer, desalination

## Abstract

The present study investigates the use of aluminum foam to enhance pure water production using a Vacuum Membrane Distillation (VMD) desalination unit. Numerical simulations were conducted for a conventional VMD and three VMD configurations with different metal foam thickness-to-channel-width ratios of h/b = (0.5, 0.75, 1). The effects of operational parameters on different VMD setups were presented and discussed. Additionally, the effects of flow rates on temperature polarization, average Nusselt number, and pressure drop were presented and discussed. The performance evaluation criterion (PEC), an indicator of the system’s global performance encompassing the heat transfer enhancement and the related pressure loss, has also been used and analyzed. Outcomes demonstrate improvements in water production with the increase in inlet velocity and temperature, while applied vacuum pressure and inlet concentration increments showed opposite behavior for all studied VMD setups. Permeate flux and temperature polarization were enhanced with metal foam insertion, and the case h = b presents the highest permeate flux and pressure drop. PEC demonstrates values superior to unity for all studied cases, with higher values for lower flow rates. Fully filled metal foam insertion is recommended for lower flow rates, while partially filled metal foam (h = 0.5b) is suggested for higher ones.

## 1. Introduction

Membrane distillation (MD) has drawn increasing attention in the last decade as a promising solution to the problems of water pollution and scarcity worldwide. MD has demonstrated outstanding potential in various separation processes, including seawater desalination and concentrated brine treatments. Membrane distillation (MD) is a hydrophobic membrane-based desalination technology for pure water separation from a hot saline solution. In MD, the temperature gradient across the hydrophobic membrane generates a vapor pressure differential. Consequently, vapor molecules migrate across the membrane from an area of higher vapor pressure to an area of lower vapor pressure.

MD presents numerous appealing properties, including low operating temperatures and highly concentrated saline solution at the inlet [1,2,3,4].

Many MD setups are considered in the literature according to how the generated vapor is recovered. Although each setup has its advantages, the vacuum membrane distillation (VMD) configuration offers increased vapor generation with decreased heat losses due to vacuum pressure applied at the permeate side [5,6]. The VMD setup is mainly used for water treatment and desalination [7,8]. Recently, VMD applications encompass the recovery of ammonia [9], acetic acid, and methanol [10], in addition to the treatment of radioactive wastewater [11].

Recent research has focused on improving VMD performance via system modifications [12,13], multistage VMD setups [14,15], and energy recovery methods [16,17]. In fact, Kotb and Khalifa [18] proposed an enhanced VMD unit operated by an air ejector and integrated with a bubble column dehumidifier. The novelty of the investigation concerns the utilization of an air ejector instead of the vacuum pump of the conventional VMD module to reduce the production cost and enable scalability for large-scale applications. They reported an enhanced performance with a productivity of 210 kg/m^2^h, at a specific energy consumption of 760 kWh/m^3^ and a gained output ratio of 0.98. The use of spacers in VMD systems to reduce polarization and improve permeate flux is investigated by Alwatban et al. [12]. They stated that the incorporation of spacers led to a reduction in concentration and temperature polarizations across the module, with larger spacer diameters contributing to improved system performance and a 53% increase in vapor flux. Anqi et al. [19] reported that introducing filaments into the VMD channel created turbulence, reducing both temperature and concentration polarization. Lee and Kim [20] developed a numerical model for a multistage VMD system, demonstrating that a 20-stage design may attain a productivity of 3.79 m^3^/day by using waste heat. Liu et al. [21] explored hybrid heat recovery methodologies in surface-heated VMD and found a 1.4-fold rise in the gained output ratio over traditional systems. Furthermore, Andr’es-Manas et al. [22] proposed a novel VMD system employing feed preheating via latent heat recovery, reaching a performance equivalent to systems with extra thermal effects but with less complexity. In addition, many studies investigated the integration of mechanical vapor recompression or solar energy in VMD [23,24]. Their findings highlighted the potential benefits of these systems.

On the other hand, it is evident that heat transfer is crucial in VMD processes. Thus, to achieve a higher permeate flux for a fixed vacuum pressure, a higher partial pressure should be produced at the membrane hot side. This pressure is directly related to the temperature of the saline solution at the membrane surface. Consequently, there is a need to improve the heat transfer at the feed side. A promising solution could be the insertion of metal foam in the feed side of the VMD module. In fact, many investigations have been conducted for heat transfer enhancement in conventional channels for cooling and heating by incorporating metal foams and have demonstrated higher heat transfer improvement [25,26,27]. In addition, a few investigations were conducted to study the effects of metal foam integration on the enhancement of MD performance. In fact, Abrofarakh [28] analyzed numerically the incorporation of metal foam in a DCMD configuration to improve its performance. The author found that the inclusion of metal foam led to a 41.5% increment in water production compared to the DCMD configuration without metal foam, with a 7.94 times decrease in entropy generation rate. In addition, Abrofarakh et al. [29] recently studied numerically an innovative approach to improve hollow-fiber DCMD by integrating copper metal foam. They demonstrated that DCMD with metal foam on both sides led to an increase in water production by up to 32% and Sherwood number by 48% compared to the typical one.

The above literature showed that the integration of metallic foam in VMD processes could be a potential modification to enhance the overall performance of the process. In this context, no numerical investigation was conducted for VMD setup enhancement by aluminum foam insertion, despite the enormous advantages of conventional VMD in comparison with other MD setups. In addition, previous studies usually used metal foam as a simple insert without focus on metal foam thickness optimization and its effects on overall VMD performance. Moreover, the previous studies investigated the thermal gain without a deep comparison with the pressure drop penalty. On the other side, Mai et al. [2] suggested in their latest review to investigate numerically new techniques to improve VMD performance with more focus on heat and mass transfer modeling. Thus, in the present study, metal foam thinness is optimized, and the performance evaluation criterion is introduced to confirm the usefulness of metal foam insertion. Additionally, the developed model is more robust and accurate because it encloses the general Darcy–Brinkman–Forchheimer model and the Navier–Stokes equations with species conservation in addition to mass and energy balances within the membrane.

Thus, the aim of this investigation is to study the performance of a conventional VMD configuration enhanced with aluminum foam inserts. The effect of aluminum foam thickness and the operational parameters on vapor generation will be studied. Furthermore, the impacts of metal foam on heat transfer, concentration and temperature polarization, pressure drop, and performance evaluation criterion (PEC) will be presented and discussed. A sensitivity analysis on the impact of key foam properties on the permeate flux and PEC is also performed.

## 2. Mathematical Model

The main assumptions considered in this study are laminar flow, incompressible saline solution, axisymmetry, constant thermophysical properties, and steady state. The metal foams are homogenous and isotropic, and the local thermal equilibrium assumption between the saline solution and the solid phase in the metal foam is considered.

**Figure 1:**
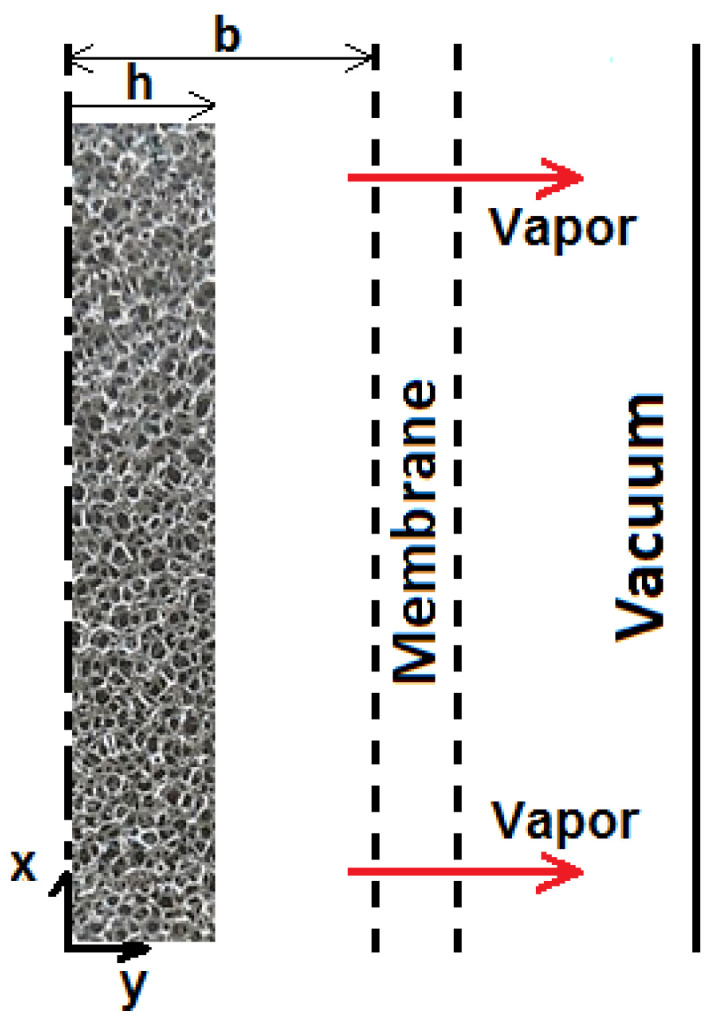
VMD setup with aluminum metal foam.

Figure 1 presents the VMD setup for saline water desalination with a PVDF hydrophobic membrane with aluminum foam inserted in the feed side. Three cases of metal foam thickness to half-channel width ratio h/b = (0.5, 0.75, 1) are investigated, as well as the conventional VMD (h/b = 0). The membrane characteristics and aluminum foam properties are presented in Table 1.

The general Darcy–Brinkman–Forchheimer model was considered in the metal foam blocks, while in the fluid region, the standard Navier–Stokes equations were adopted. In this study, a general system of conservation equations will be considered for both the clear solution and the aluminum foam-solution matrix. Thus, the equations of continuity, momentum, species, and energy based on the local thermal equilibrium assumption between the fluid and solid phases in the metal foam are as follows [32,33,34]:

(1) $$\frac{\partial U}{\partial x}+\frac{\partial V}{\partial y}=0$$

(2) $$U\frac{\partial U}{\partial x}+V\frac{\partial U}{\partial y}=-\frac{\varepsilon^{2}}{\rho_{f}}\frac{\partial P}{\partial x}+\frac{\varepsilon \mu }{\rho_{f}}\left(\frac{\partial^{2}U}{\partial x^{2}}+\frac{\partial^{2}U}{\partial y^{2}}\right)-B\frac{\varepsilon^{2}}{\rho_{f}}\left(\frac{\mu }{K}U+\frac{\rho_{f}}{\sqrt{K}}F\left|\vec{u}\right|U\right)$$

(3) $$U\frac{\partial V}{\partial x}+V\frac{\partial V}{\partial y}=-\frac{\varepsilon^{2}}{\rho_{f}}\frac{\partial P}{\partial y}+\frac{\varepsilon \mu }{\rho_{f}}\left(\frac{\partial^{2}V}{\partial x^{2}}+\frac{\partial^{2}V}{\partial y^{2}}\right)-B\frac{\varepsilon^{2}}{\rho_{f}}\left(\frac{\mu }{K}V+\frac{\rho_{f}}{\sqrt{K}}F\left|\vec{u}\right|V\right)$$

(4) $$U\frac{\partial T}{\partial x}+V\frac{\partial T}{\partial y}=\frac{k_{eff}}{\rho C_{p}}\left(\frac{\partial^{2}T}{\partial x^{2}}+\frac{\partial^{2}T}{\partial y^{2}}\right)$$

(5) $$U\frac{\partial C}{\partial x}+V\frac{\partial C}{\partial y}=\varepsilon D\left(\frac{\partial^{2}C}{\partial x^{2}}+\frac{\partial^{2}C}{\partial y^{2}}\right)$$

where

(6) $$k_{eff}=\left(1-\varepsilon \right)k_{s}+{\varepsilon k}_{f}$$

(7) $$\left|\vec{u}\right|=\sqrt{U^{2}+V^{2}}$$

(8) $$F=\frac{1.75}{\varepsilon \sqrt{150}}$$

*U*, *V*, *P*, *C*, *μ*, *K*, *ε*, *ρ*, *F*, *T*, and *C_p_* represent the axial velocity, the radial velocity, pressure, mass fraction of NaCl, viscosity, metal foam permeability, metal foam porosity, density, Forchheimer coefficient, temperature, and specific heat, respectively. In the fluid zone, parameter *B* is equal to 0 and *ε* = 1. In addition, the effective thermal conductivity (*k_eff_*) in the fluid zone is equal to the fluid thermal conductivity (*k_f_*). In the metal foam layer, *B* is equal to 1, and *ε* is equal to the porosity of the metal foam [33,34].

The boundary conditions are the following:

(9) $$\mathrm{At}\;x=0:\;U=U_{in},\;V=0,\;T=\;T_{in},\;C=C_{in}$$

(10) $$\mathrm{At}\;y=0:\frac{\partial U}{\partial y}=0,\;\frac{\partial T}{\partial y}=0,\;\frac{\partial C}{\partial y}=0,\;V=0$$

(11) $$\mathrm{At}\;x=L:\;\frac{\partial U}{\partial x}=0,\;\frac{\partial V}{\partial x}=0,\;\frac{\partial T}{\partial x}=0,\;\frac{\partial C}{\partial x}=0$$

(12) $$\mathrm{At}\;y=b:\;U=0,\;V=\frac{J_{v}}{\rho },\;\frac{\partial T}{\partial y}=-\frac{Q_{c}+Q_{L}}{k},\;\frac{\partial C}{\partial y}=\frac{J_{v}}{\rho D}$$

(13) $$\mathrm{At}\;y=b+\delta :\;P=P_{vc}$$

where *J_v_*, *Q_L_*, and *Q_c_* represent, respectively, the local vapor flux, the latent, and the conduction heat flux [35,36,37].

(14) $$J_{v}=K_{m}\left(P_{hm}-P_{vc}\right)=1.064\frac{\gamma r_{p}}{\tau \delta }{\left(\frac{M}{RT}\right)}^{0.5}\left(P_{hm}-P_{vc}\right)$$

The vapor pressure at the hot side *P_hm_* is expressed as follows [37]:

(15) $$P_{hm}=\left(1-C_{M}\right)exp\left(23.1964-\frac{3816.44}{T_{hm}-46.13}\right)$$

The averaged vapor flux is defined as follows [38]:

(16) $$J=\frac{1}{L}\int_{0}^{L}J_{v}\left(x\right)dx$$

On the other side, the temperature polarization coefficient (*TPC*) is calculated using Equation [39]:

(17) $$TPC=\frac{T_{hm}-T_{vc}}{T_{b}-T_{vc}}$$

The concentration polarization coefficient (*CPC*) is defined as the ratio of the concentration at the membrane interface (*C_hm_*) to the concentration in the bulk solution (*C_b_*), which is given as follows [40]:

(18) $$CPC=\frac{C_{hm}}{C_{b}}$$

The heat transfer enhancement ratio (*HTER*) is defined as the heat transfer with the insertion of metallic foams to heat transfer within a clear tube, which is calculated using Equation (19) [41,42,43]:

(19) $$HTER=\frac{{Nu}_{avg,metal\;foam}}{{Nu}_{avg,\;clear\;tube}}$$

The performance evaluation criterion (PEC) is an indicator of the global performance of the heat exchanger. In general, PEC represents the ratio between the rate of heat transfer enhancement after making modifications to the rate of pressure losses resulting from the modifications. The modification in the current study is the insertion of the metallic foam. If the PEC value is greater than unity, it means that the modifications are beneficial. The PEC is calculated by Equation (20) [32,41]:

(20) $$PEC=\frac{\frac{{Nu}_{avg,\;metal\;foam}}{{Nu}_{avg,\;clear\;tube}}}{{\left(\frac{f_{metal\;foam}}{f_{metal\;clear\;tube}}\right)}^{1/3}}$$

where *f* is the friction factor, and Nu is the Nusselt number.

All results achieved in this investigation were calculated under the following conditions: *P_vc_* = 5 kPa, b = 3 mm, *T_in_* = 55 °C, *U_in_* = 0.15 m/s, *L* = 30 cm, *C_in_* = 0.035.

## 3. Numerical Method and Validation

The Simpler Algorithm and the finite volume approach are used to solve the developed model [42]. The solution approach was subjected to a grid-dependence analysis, as shown in Table 2. One can see that the values are unaffected by the grid size. Therefore, a 700 × 40 grid is taken into consideration for all calculations presented in this study. All simulations assume a structured quadrilateral mesh with boundary layer refining near the wall. The calculations are stopped once the maximum absolute mass residual values in the control volume and the overall domain are smaller than 10^−6^.

**Figure 2:**
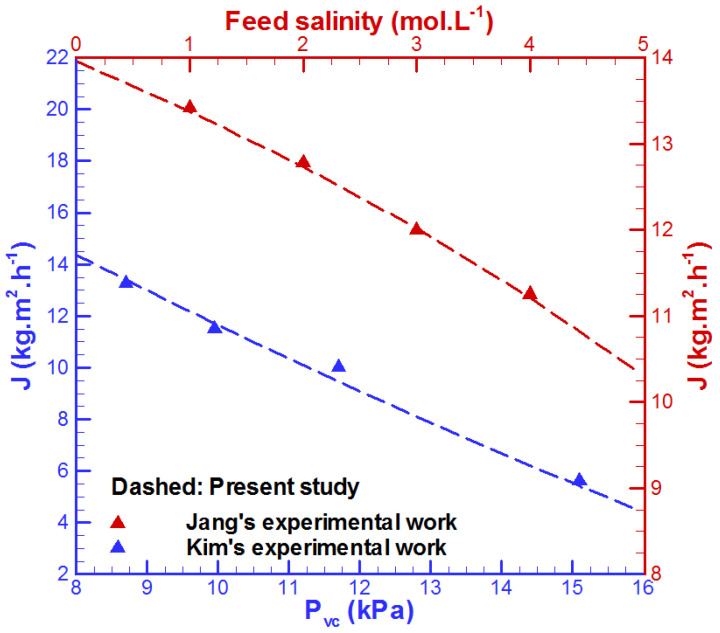
Comparison of the developed model with experimental data (Kim et al. [43] and Jang et al. [44]).

**Figure 3:**
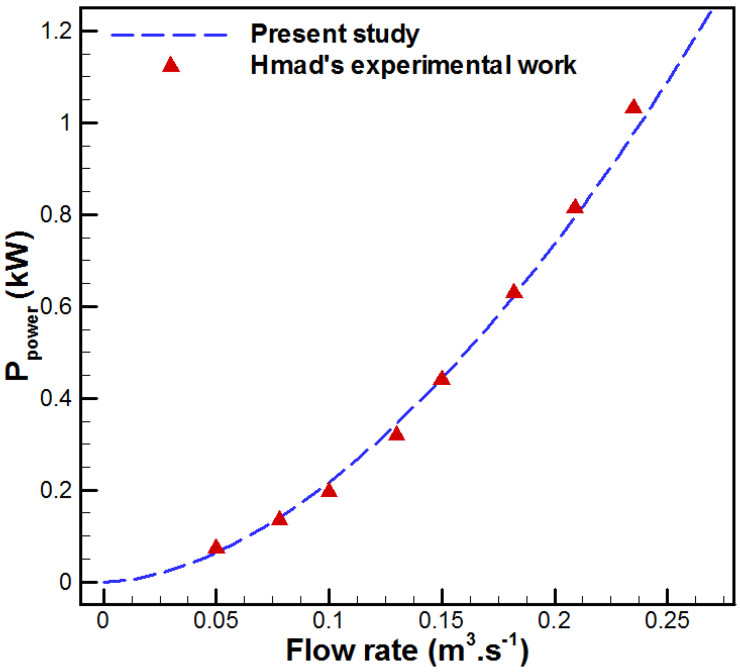
Comparison of the developed model with experimental data (Hmad et al. [45]).

The validation of the model is conducted with respect to the experimental results provided by Kim et al. [43] and Jang et al. [44] related to a VMD unit and the data provided by Hmad et al. [45] for a flow in a channel with metal foams. As shown in Figure 2 and Figure 3, the present model is accurate. In fact, the average relative errors for permeate flux between the developed model and experimental results achieved by Kim et al. [43] and Jang et al. [44] are 3.36% and 1.12%, respectively. In addition, the average relative error for pumping power between the developed model and Hmad et al. [45] experimental data is 4.62%.

In addition, the root mean square error (*RMSE*) is employed for better evaluation of the error between the present model and experimental data [28].

(21) $$RMSE=\sqrt{\frac{1}{n}\sum_{i=1}^{n}{\left(y_{expermental\_i}-y_{numerical\_i}\right)}^{2}}$$

The *RMSE* for water production between the present model and experimental data provided by Kim et al. [43] and Jang et al. [44] are 0.351 kg/m^2^h and 0.163 kg/m^2^h, respectively. Additionally, the *RMSE* of pumping power is 0.0198 kW. Thus, the developed model is accurate for data prediction.

## 4. Results and Discussion

We started the investigation by studying the effects of the operational parameters corresponding to the inlet velocity (*U_in_*), inlet temperature (*T_in_*), inlet concentration (*C_in_*), and vacuum pressure (*P_vc_*) on vapor production (*J*). The four VMD configurations, h/b = (0, 0.5, 0.75, 1), are compared under the same operating conditions, where h/b = 0 corresponds to the conventional VMD without metal foam and h/b = 1 represents the fully filled aluminum foam VMD.

**Figure 4:**
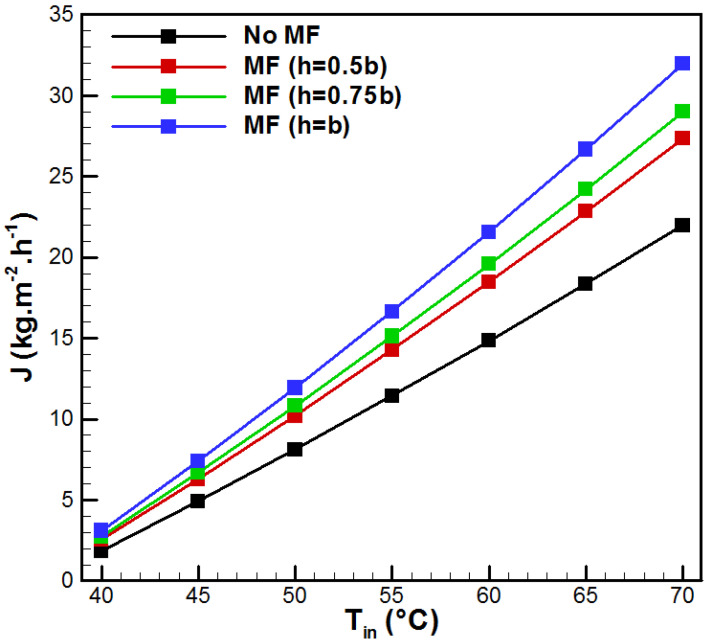
Evolution of vapor flux with *T_in_*.

Figure 4 presents the effects of inlet temperature on vapor generation (*J*) for the different VMD cases. In fact, increasing *T_in_* from 40 to 70 °C makes *J* increase 11.94, 10.75, 10.53, and 10.27 times for the conventional VMD, h = 0.5b, h = 0.75b, and h = b, respectively. These increments are evidenced by the rise in vapor pressure at the hot membrane side, leading to an increase in the driving force, and, consequently, more permeate flux is generated at the vacuum side. Therefore, the incorporation of metal foam ensures more water production in comparison with conventional VMD. This increase is proportional to the metal foam thickness. Thus, for *T_in_* = 55 °C, increments of 24.81%, 32.19%, and 45.49% occurred for h = 0.5b, h = 0.75b, and h = b, respectively. This enhancement is because of aluminum foam insertions, which are highly permeable porous structures. In fact, when fluids flow through the metal foam, the saline solution is permanently forced to change direction with increasing velocity gradients in the open-cell spaces. Thus, the thermal boundary layers are thinner and repeatedly disturbed. Consequently, the better flow mixing and the improvements in overall transport phenomena across the membrane lead to higher vapor generation.

**Figure 5:**
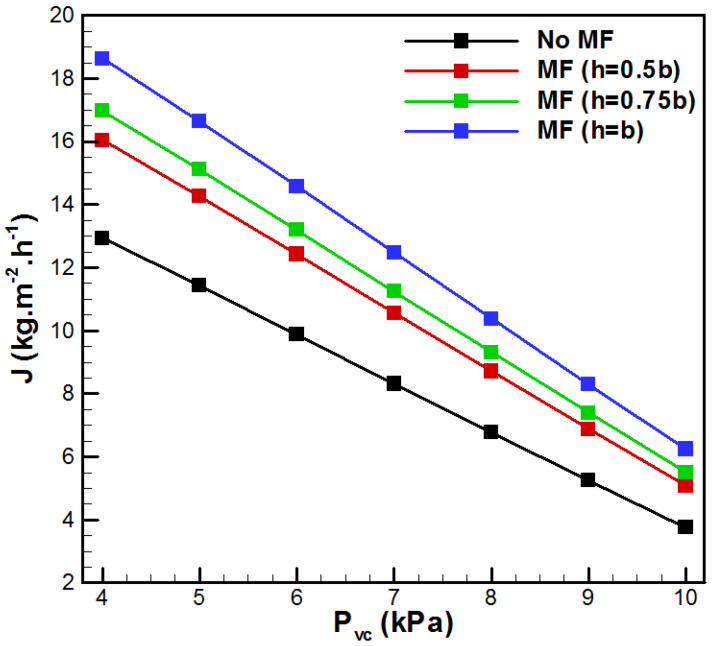
Evolution of vapor flux with *P_vc_*.

The variation in vapor flux with the permeate side pressure for different metal foam thicknesses is presented in Figure 5. It is important to mention that vacuum pressure is a specific operational parameter for the VMD process. In fact, a decrease in *P_vc_* induces increments in vapor flux for all the VMD cases. The decrease in *P_vc_* from 10 kPa to 4 kPa results in an increase in J by 3.44, 3.15, 3.08, and 2.98 times, respectively, for the cases h = 0, h = 0.5b, h = 0.75b, and h = b. The increase in vapor production with the decrease in the vacuum pressure is because of the rise in the VMD driving force. In addition, one can see that the incorporation of metal foams in the VMD feed side enhanced *J*, and this increase appears to be more pronounced for low vacuum pressures. The case with h = b (fully filled with metal foam) produces the highest *J* for all vacuum pressures and reaches 66.5% enhancement for *P_vc_* = 10 kPa.

In fact, the fully filled VMD case is subjected to better fluid mixing and thinner boundary layers with better heat transfer from the channel’s center to the vicinity of the membrane due to the incorporation of aluminum metal foam. In this case, mass transfer resistance is reduced at the membrane side, and driving force is amplified as described by Equations (14) and (15).

**Figure 6:**
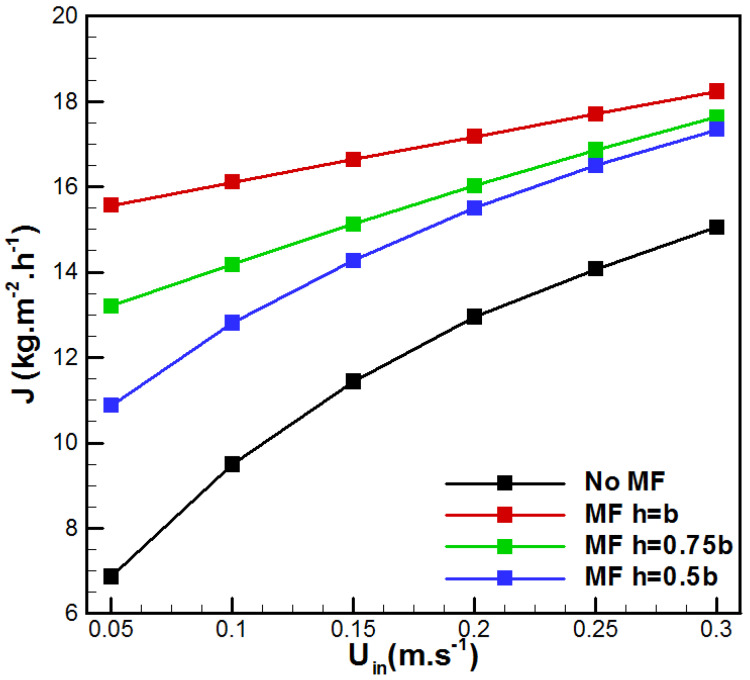
Evolution of vapor flux with *U_in_*.

The evolution of the water production *J* with the inlet velocity *U_in_* is presented in Figure 6. As expected, increasing the flow rate also increases the pure water production for both cases with and without the inserted metal foam. In fact, for *U_in_* = 0.05 m/s, the insertion of metal foam improved *J* by 58.45%, 92.41%, and 126.82%, respectively, for the cases h = 0.5b, h = 0.75b, and h = b, in comparison with the VMD process without metal foam. The increase in flow rate with the incorporation of metal foam leads to an enhancement in heat transfer and makes the hot side membrane surface’s temperature approach the bulk temperature, and, consequently, a rise in the vapor driving force is obtained. In addition, it is important to note that adding metal foams makes the vapor generation and transport less sensitive to the inlet feed velocity.

**Figure 7:**
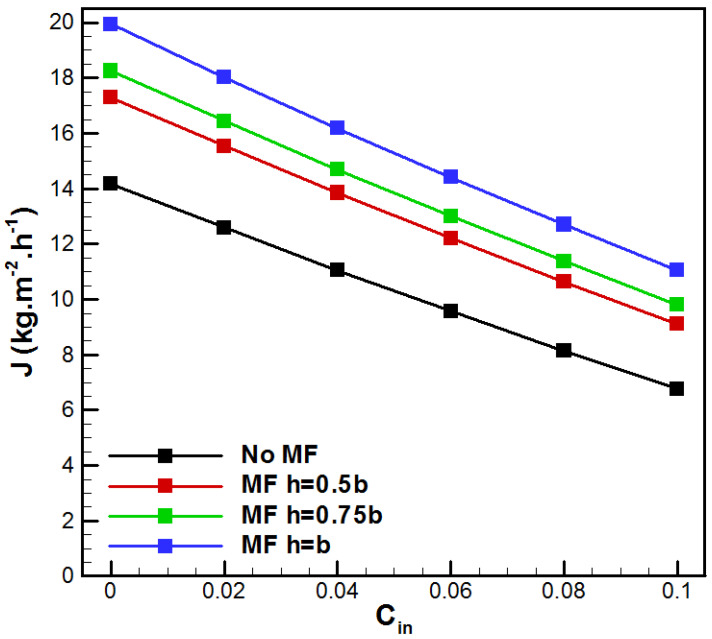
Evolution of vapor flux with *C_in_*.

Figure 7 presents the effect of the saline solution inlet concentration *C_in_* on the permeate flux for the different VMD cases. Higher saline solution concentration induced lower water production for all studied cases because a rise in *C_in_* reduces the partial vapor pressure of water, leading to a drop in the driving force. In addition, for *C_in_* = 0.1, higher values of metal foam thickness make *J* increase by 34.76%, 45.11%, and 63.41%, respectively, for the case h/b = (0.5, 0.75, 1), in comparison with conventional VMD.

**Figure 8:**
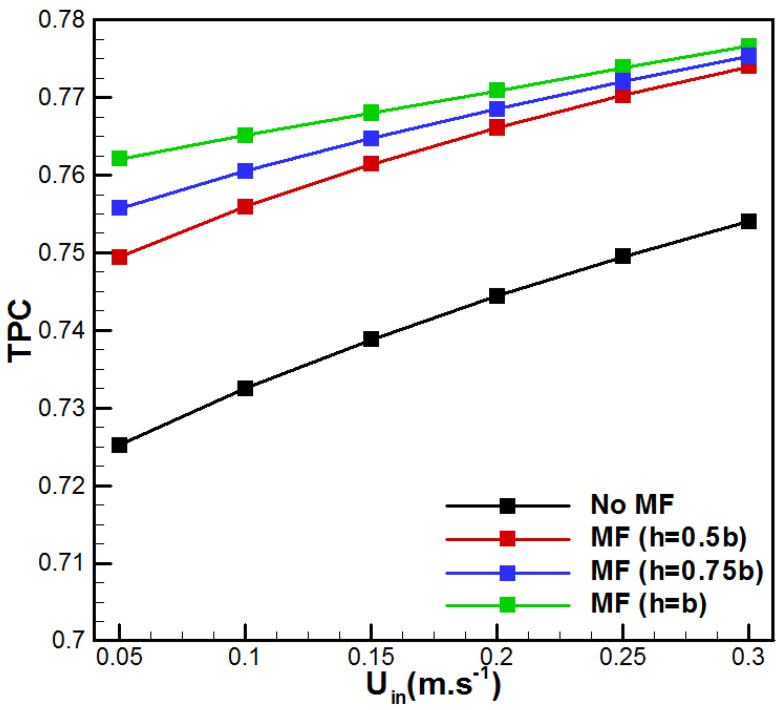
Evolution of *TPC* with inlet velocity.

The variation in temperature polarization with flow rate for the VMD-studied cases is presented in Figure 8. It is good to remind that temperature polarization near the hot membrane surface is a major reason for the declination of permeate flux in the VMD process. In fact, as *TPC* approaches unity, the effect of temperature polarization becomes insignificant, since the interface temperature becomes very close to the saline solution bulk temperature [46]. From Figure 8, it is clear that higher inlet velocities make *TPC* increase for the studied VMD. In addition, the insertion of metal foam promotes higher temperature polarization coefficients. Thus, the combination of metal foam with high flow rates reduces the temperature polarization effects due to the proper mixing within the boundary layer and encourages hot bulk liquid to reach the membrane surface due to the combined effects of conduction through foam material and liquid forced convection. In fact, the metal ligaments acting like microfins enable the compensation of heat loss during evaporation through conduction from the center of the channel to the vicinity of the membrane. On the other side, aluminum foam supports convective mixing due to increments in velocity gradients across open-cell spaces. Consequently, there is a more uniform temperature at the membrane feed side and fewer temperature polarization effects.

**Figure 9:**
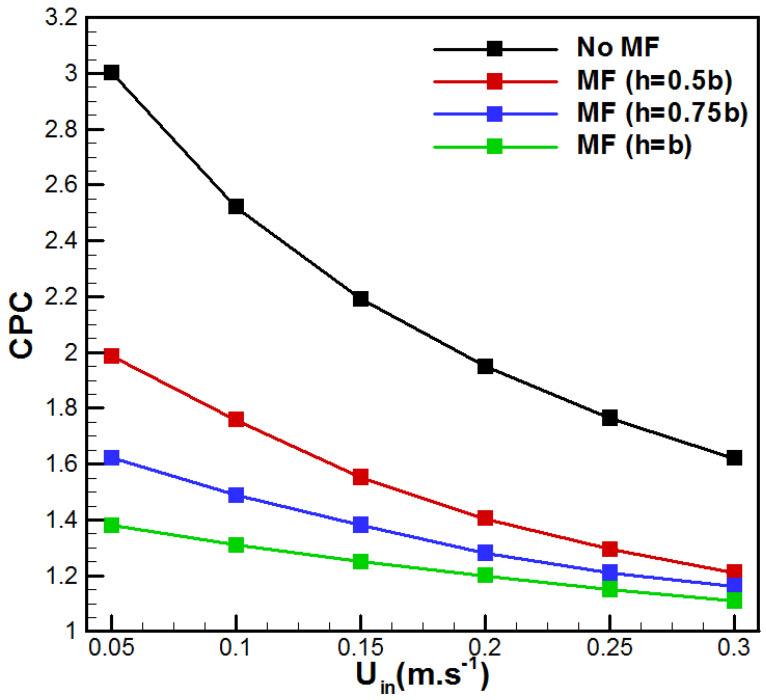
Evolution of *CPC* with inlet velocity.

Additionally, the effect of metal foam addition on the evolution of the concentration polarization coefficient (*CPC*) is illustrated in Figure 9. As expected, higher flow rates lead to a reduction in the concentration polarization effects. Furthermore, the addition of metal foam promotes a reduction in *CPC* depending on its thickness. In fact, metal foam integration leads to a reduction in the concentration boundary layer thickness because of an increment in mixing, which in turn results in lower *CPC*. Thus, VMD enhanced with metal foam integration achieved lower mass transfer resistance, uniform distribution of salt concentration, and consequently higher evaporation rates.

**Figure 10:**
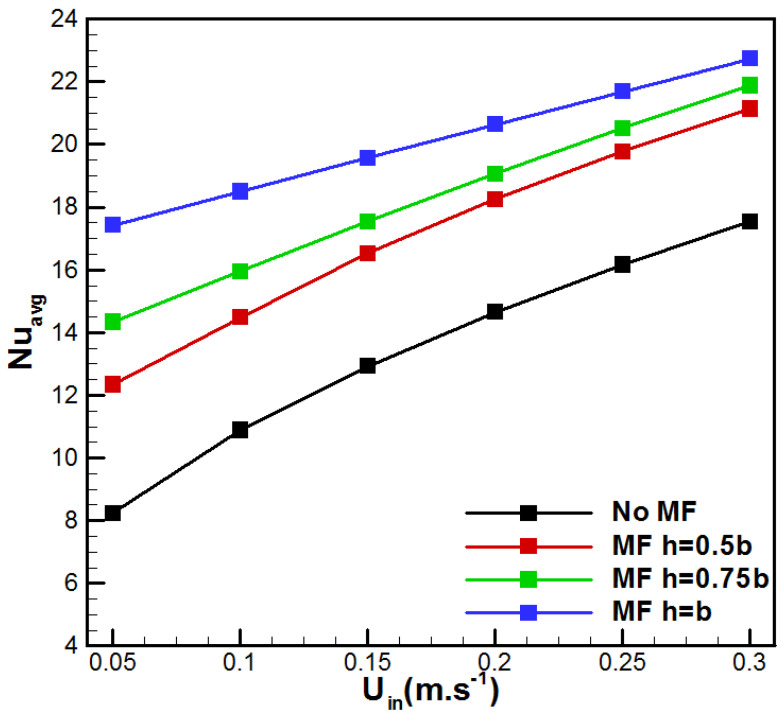
Effect of *U_in_* on the average Nusselt number.

**Figure 11:**
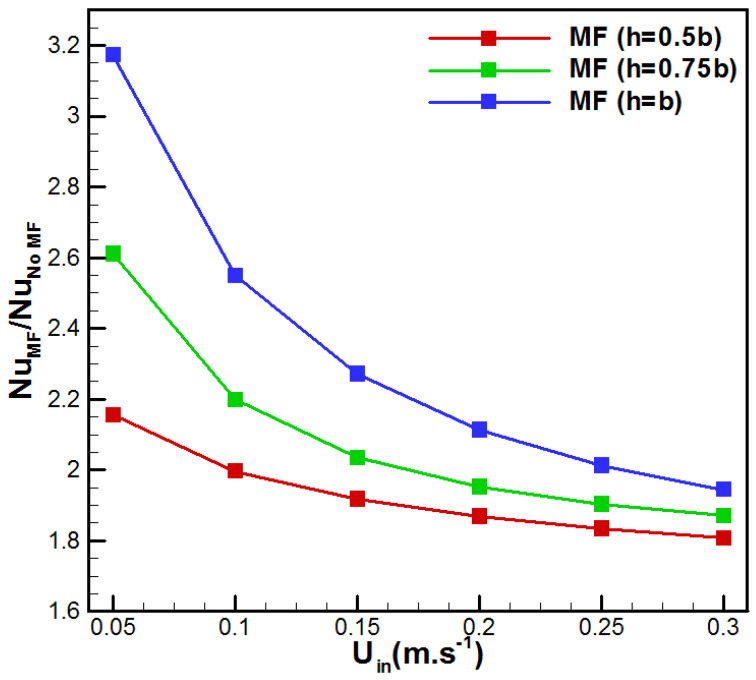
Effect of *U_in_* on the heat transfer enhancement ratio.

The variation in the average Nusselt number with feed velocity for the considered VMD configurations is depicted in Figure 10. Figure 10 demonstrates the combined effects of feed velocity and metal foam insertion on the evolution of the average Nusselt number for the cases h/b = (0, 0.5, 0.75, 1). One can see that the convective heat transfer increases with feed velocity for all VMD cases. The VMD case with h = b demonstrated the highest value of Nuavg, while the conventional VMD (h/b = 0) demonstrated the minimum one. In addition, from the heat transfer enhancement ratio presented in Figure 11, one can see that for *U_in_* = 0.05 m/s, *Nu_avg_* increased by 3.17, 2.61, and 2.15 times, respectively, for the cases h/b = (0, 0.5, 0.75, 1). In fact, these improvements result from the enhancement in the heat transfer due to metal foam insertion. On the other side, higher velocities lead to lower enhancement ratios. Such an observation may be attributed to the reduced residence time in the foam for the faster feed solution.

**Figure 12:**
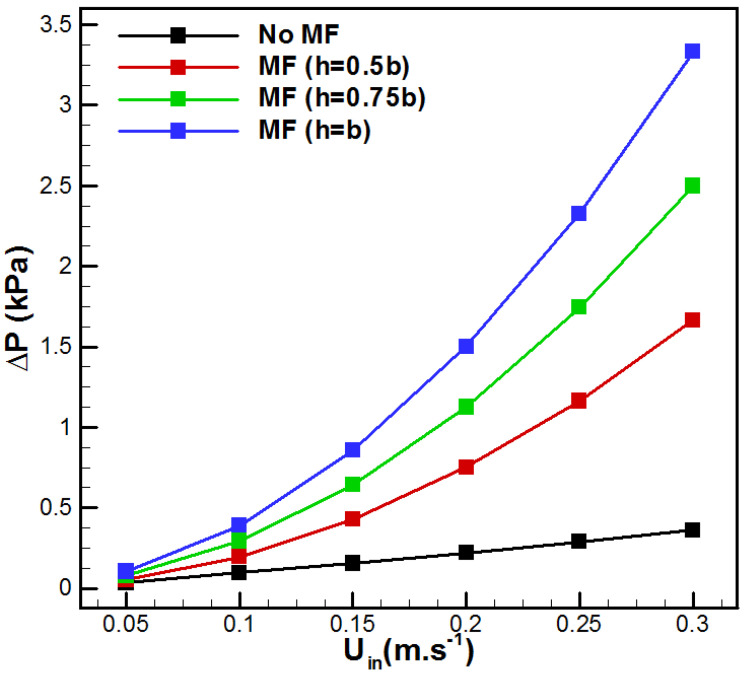
Effect of *U_in_* on the pressure drop.

**Figure 13:**
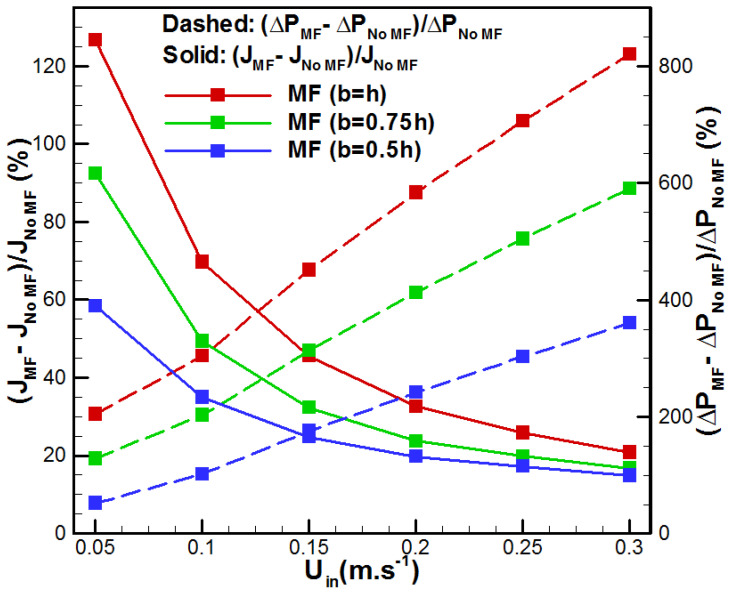
Effect of *U_in_* on permeate flux gain and pressure drop penalty.

The above, Figure 10 and Figure 11, highlight a general trend characterized by an evident heat transfer enhancement associated with the presence of the metal foam in the feed solution channel. However, the overall performance also depends on the supplied pumping power and related pressure losses due to the metal foam insertion. Figure 12 depicts the variation in the pressure drop with the flow rate for the studied cases. One can see that the insertion of metal foam raises the pressure drop in comparison with the conventional VMD. In fact, additional pressure drop occurred depending on the metal foam thickness on the feed side. The metal foam totally filled case (h = b) showed the highest ΔP with an increase of 820.59% when *U_in_* = 0.3 m/s, in comparison with the no metal foam case, while the cases h = 0.75b and h = 0.5b presented an increase of 590.44% and 360.29%, respectively. This order of magnitude for pressure drop variation was recently reported by Abrofarakh [29]. At lower velocity (*U_in_* = 0.05 m/s), the pressure drop increases by 18.18%, 77.24%, and 136.32%, respectively, for the cases h = 0.5b, h = 0.75b, and h = b. In fact, saline solution in the metallic foam matrix faces a lot of obstructions and redirections through tortuous paths, leading to additional friction and significant momentum losses. In addition, Figure 13 shows the variation in vapor flux gain and pressure drop penalty with saline solution inlet velocity. It resumed the gain in permeate flux with the pressure drop penalty due to metal foam insertion. In fact, with higher flow rates, permeate flux gain reduces while pressure drop penalty increases for all cases of metal foam incorporation. In addition, fully filled metal foam VMD (h = b) depicts the highest permeate flux gain and pressure drop penalty. Furthermore, *U_in_* = 0.05 m/s demonstrates the highest permeate flux gain with the lowest pressure drop penalty, while *U_in_* = 0.3 m/s presents the lowest permeate flux gain and the highest pressure drop penalty. Thus, better results are attributed to lower flow rates.

From the previous results, one can conclude that the insertion of metal foam in a conventional VMD process significantly enhanced heat transfer and consequently allowed higher pure water production with an increase in pressure drop. However, circulating the feed saline solution at low velocities considerably enhances the heat transfer while keeping the pressure loss cost reduced. Therefore, from an experimental implementation view, it is recommended to operate at lower inlet velocities.

On the other side, the temperature gain and extra pressure loss due to metal foam insertion raise the question of whether metal foams are beneficial for conventional VMD. To respond to this question, the performance evaluation criterion (*PEC*) can be used to recommend or not the use of these metal foams for higher water production and higher VMD performance in general. If the modification has a *PEC* greater than unity, it will be adopted as a useful modification; otherwise, the heat transfer gain is less than the extra pressure loss, and thus the aluminum foam insertion is not cost-effective.

**Figure 14:**
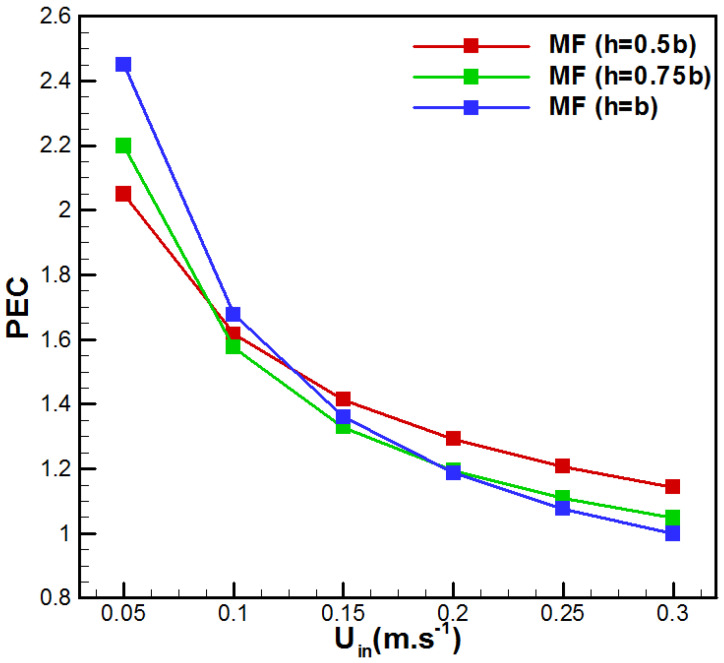
Effect of *U_in_* on the performance evaluation criterion.

Therefore, Figure 14 presents the evolution of *PEC* as a function of the inlet velocity for the different metal foam cases.

It is important to notice that all metal foam thicknesses display a *PEC* superior to unity. PEC is higher at low flow rates and decreases with an increase in *U_in_*. Thus, fully filled aluminum foam VMD is recommended for lower flow velocity to display higher efficiency. For higher velocities, partial metal foam insertion with h = 0.5b presents better efficiency. A more detailed analysis of the results depicted in Figure 14 shows a change in behavior of the *PEC* with *U_in_*. The totally filled option (b = h) has a higher *PEC* than the two other cases for lower inlet saline solutions. The reverse case is observed for a faster feed solution. On the other side, as depicted in Figure 12, the maximum pressure drop corresponds to the case with h = b and *U_in_* = 0.3 m/s. In this scenario, the pressure drop ΔP is 3.3325 kPa, whereas the case without metal foam at the same flow rate shows a pressure drop of 0.362 kPa. Even though the increase in pressure drop is equal to 820.59%, the incremental pumping power corresponds to Pp = ΔP·Q = 0.107 W. This value is negligible compared with commonly used membrane desalination processes such as reverse osmosis. Additionally, the highest feed-side pressure drop in the proposed design is far lower than usually reported DCMD values (ΔP = 21.2 kPa at Re = 1500) [47]. One can conclude that the highest hydraulic penalty due to metal foam insertion is realistic and industrially achievable. In addition, for scale-up, it is recommended to use many parallel channels with removable aluminum foam for inspection and cleaning.

On the other side, the impact of key foam properties, such as porosity (ε) and pore density (PPI), on the permeate flux and *PEC* could be achieved by a sensitivity analysis approach.

In fact, sensitivity analysis is used to investigate the influence of input variables on outputs. The inputs are PPI and porosity of metal foam, while the outputs are permeate flux (*J*) and performance evaluation criterion (*PEC*). Thus, input parameters are perturbed, and their impacts on outputs are calculated as follows [48,49]:

(22) $${SS}_{IJ}=\frac{\partial Y_{i}}{\partial R_{j}}\frac{R_{j}}{Y_{i}}$$

where *SS_IJ_* is the normalized local sensitivity coefficient, and *Y_i_* and *R_j_* are the i^th^ output and the j^th^ input, respectively.

**Figure 15:**
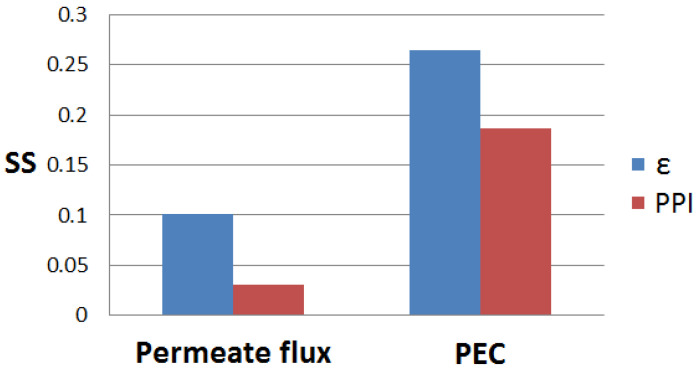
Sensitivity analysis for *J* and *PEC*.

From Figure 15, the calculation of the normalized local sensitivity coefficients revealed that a 1% change in porosity leads to a 0.1% change in permeate flux (*J*) and 0.264% in performance evaluation criterion (*PEC*). On the other side, a 1% change in PPI makes the permeate flux change by 0.031% and *PEC* by 0.187%. One can conclude that *J* and *PEC* are more sensitive to changes in porosity than to changes in PPI.

## 5. Conclusions

This study concerns a numerical study on the enhancement of pure water production generated by a conventional VMD process through the insertion of aluminum foam with different thickness-to-channel-width ratios. The numerical model was systematically checked and validated with experimental data from the literature. Results demonstrate improvement in water production with the increase in inlet velocity and temperature, while applied vacuum pressure and inlet concentration rise showed opposite behavior for all studied VMD configurations. Significant water production enhancement was observed for the case with metal foam insertion, and particularly the fully filled metal foam VMD (h = b) with low feed solution inlet velocity. Permeate flux increment depends on operational parameters and reached 126.82% for *U_in_* = 0.05 m/s, *P_vc_* = 5 kPa, *T_in_* = 55 °C, and *C_in_* = 0.035. Temperature polarization effects were improved with metal foam insertion, with a considerable increase in the Nusselt number, with a heat transfer enhancement ratio reaching 3.17 for fully filled metal foam VMD at *U_in_* = 0.05 m/s. Pressure drop exhibits opposite behavior with metal insertion, with higher values at *U_in_* = 0.3 m/s. The performance enhancement criterion (*PEC*) recommended the incorporation of a fully filled metal foam in a VMD module for lower velocity applications and partially filled metal foam (h = 0.5b) for higher flow rates. Further studies on this subject, considering second law analysis and optimization techniques, are under development. The sensitivity analysis results demonstrate that the permeate flux and performance evaluation criterion are more sensitive to changes in porosity than to changes in pore density.

## Figures and Tables

**Table 1:** Aluminum foam and PVDF membrane characteristics.

Membrane	PVDF [30]	Metal Foam	Aluminum [31]
Nominal pore size (μm)	0.22	Pores per inch, PPI	45
Thickness (μm)	125	Porosity	0.9
Porosity	0.75	Permeability K (m^2^)	0.420 × 10^−7^

**Table 2:** Impact of grid size on the vapor flux and the average Nusselt number.

Nx, Ny	700, 40	800, 40	700, 50	800, 50
J [kg/m^2^h]	17.110	17.111	17.110	17.112
*Nu_avg_*	10.228	10.231	10.229	10.230

## Data Availability

The original contributions presented in this study are included in the article. Further inquiries can be directed to the corresponding author(s).
